# Controlling the Wettability of ZnO Thin Films by Spray Pyrolysis for Photocatalytic Applications

**DOI:** 10.3390/ma15093364

**Published:** 2022-05-07

**Authors:** Muhammad Rabeel, Sofia Javed, Ramsha Khan, Muhammad Aftab Akram, Shania Rehman, Deok-kee Kim, Muhammad Farooq Khan

**Affiliations:** 1 Nanomaterials Synthesis Laboratory, School of Chemical and Materials Engineering, National University of Sciences and Technology, H-12, Islamabad 44000, Pakistan; rana.rabeel12@gmail.com (M.R.); khanramsha94@gmail.com (R.K.); aftabakram@scme.nust.edu.pk (M.A.A.); 2 Department of Electrical Engineering, Sejong University, 209 Neungdong-ro, Gwangjin-gu, Seoul 05006, Korea; shania.rehman19@gmail.com

**Keywords:** zinc oxide thin films, spray pyrolysis, contact angle, photocatalysis, rhodamine B, wettability

## Abstract

Herein, we synthesized the zinc oxide (ZnO) thin films (TFs) deposited on glass substrates via spray pyrolysis (SP) to prepare self-cleaning glass. Various process parameters were used to optimize photocatalytic performance. Substrates were coated at room temperature (RT) and 250 °C with a 1 mL or 2 mL ZnO solution while maintaining a distance from the spray gun to the substrate of 20 cm or 30 cm. Several characterization techniques, i.e., XRD, SEM, AFM, and UV–Vis were used to determine the structural, morphological, and optical characteristics of the prepared samples. The wettability of the samples was evaluated using contact angle measurements. As ZnO is hydrophilic in nature, the RT deposited samples showed a hydrophilic character, whereas the ZnO TFs deposited at 250 °C demonstrated a hydrophobic character. The XRD results showed a higher degree of crystallinity for samples deposited on heated substrates. Because of this higher crystallinity, the surface energy decreased, and the contact angle increased. Moreover, by using 2 mL solution, better surface coverage and roughness were obtained for the ZnO TFs. However, by exploiting the distance of the spray to the samples size distribution and surface coverage can be controlled, the samples deposited at 30 mL showed a uniform particle size distribution from 30–40 nm. In addition, the photoactivity of the samples was tested by the degradation of rhodamine B dye. Substrates prepared with a 2 mL solution sprayed at 20 cm showed higher dye degradation than other samples, which can play a vital role in self-cleaning. Hence, by changing the said parameters, the ZnO thin film properties on glass substrates were optimized for self-cleaning diversity.

## 1. Introduction

Due to the significant greenhouse effect and environmental degradation, a switch to sustainable and green energy resources is urgently required [1]. Self-cleaning is the most attractive area of research due to its wider applications. Self-cleaning coatings based on thin films or nanoparticle semiconductors have grown in popularity over the last decade [2,3,4,5] owing to their commercialization prospects and wide range of applications in window glass [6,7], fabrics [8], paints [9], construction materials, plastics [10], corrosion protection [11], and solar cells [12]. Self-cleaning can be achieved by changing the surface wettability properties so that the surfaces can be optimized to make them hydrophobic and hydrophilic. Surfaces with exceptional wettability have been created utilizing a variety of materials [13]. The self-cleaning property is achieved by two mechanisms that include the spreading of water, which is the sheeting effect, and photocatalysis [3].

Many semiconductors are promising as prospective photocatalysts, such as VO_3_ [10], ZnS, CdS [14], and ZnO, which are leading the way [15,16,17,18,19,20,21]. ZnO is also a non-toxic wide bandgap semiconductor with good thermal and mechanical stability, and strong photocatalytic activity [22]. The development of semiconductor (SC) metal oxide thin films of tunable bandgap, with various morphologies and self-cleaning attributes, are the key for optoelectronic applications. ZnO has been one of the most widely studied semiconductors with various applications in photovoltaics and as a photocatalyst [23,24,25].

Several techniques have been adopted to synthesize the ZnO thin films (TFs) [26,27,28,29], most of which require a high vacuum, high temperature, costly equipment, catalysts, and the use of toxic gas compounds. As compared to other synthesis methods, the spray pyrolysis technique (SPT) is efficient and relatively low-cost for the fabrication of ZnO TFs [30]. This technique provides better control over the morphology and growth orientation of ZnO TFs by adjusting the process parameters [31].

Hunge et al. [32] prepared multifunctional ZnO TFs by SPT; they varied the substrate temperature while keeping all other parameters fixed and studied the devices for photo electrocatalytic degradation and gas sensing. Tarwal et al. [33] prepared ZnO TFs by SPT, and they varied the solution concentration and studied their effect on wetting properties. Ravichandran et al. [34] prepared ZnO TFs by SPT with various film thicknesses and studied their photocatalytic effect. Antar et al. [35] showed the facile synthesis of ZnO TFs by SPT. Islam et al. [36] showed the photocatalytic activity of ZnO TFs. Zirak et al. [37] prepared composite thin films of ZnO and carbon quantum dots and used various process parameters. However, the comparative variations in the volume of solution, the distance between the spray gun and substrate, and the substrate temperature have not yet been reported all together for wettability and photocatalytic applications.

The focus of the present study is therefore to prepare and optimize ZnO TFs for wettability and self-cleaning applications using different optimization parameters and studying their effects using SPT. The samples were prepared by SPT by varying the distance of the spray gun to the sample, temperature of the substrate, and volume of the ZnO solution.

## 2. Materials and Methods

The soda–lime glass substrates were first cut 2 × 2 cm^2^ and then cleaned by sonicating them in deionized water for 10 min and then by sonicating in ethanol for 10 min. They were further rinsed using acetone and isopropyl alcohol (IPA) to obtain clean surfaces for the coatings. Then they were dried in a lab oven for 10 min at 100 °C.

The solution was prepared using zinc acetate dehydrate, isopropyl alcohol (IPA), and monoethanolamine (MEA) purchased from Sigma-Aldrich. All the reagents were of analytical grade with >99% purity. The zinc acetate dehydrate was used as a zinc precursor, whereas the MEA was used as a stabilizing agent for the ZnO solution preparation. A 0.2 M solution was prepared by dissolving the zinc acetate dehydrate in IPA with dropwise addition of MEA. Magnetic stirring at 60 °C was applied throughout the process until the transparent solution was obtained. This solution was then left for aging for 24 h [38].

**Figure 1:**
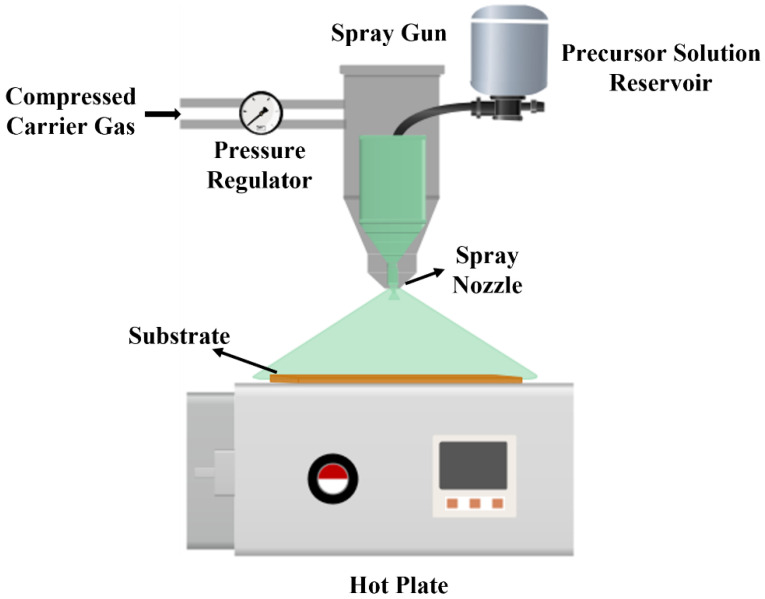
Schematic of Spray Pyrolysis Setup.

The ZnO solution was deposited over the pre-cleaned soda–lime glass substrates. The SPT setup was used for the depositions, whose schematic is shown in Figure 1. The spray pyrolysis was conducted using an aluminum alloy cup having a capacity of 25 mL with a spray width of 50 mm. The nozzle caliber was 0.5 mm. The coatings were carried out using different parameters, i.e., deposition temperature, the distance of the spray gun to the substrate, and volume of the solution to optimize thin films with better self-cleaning and photocatalytic properties. The coatings were made at two different temperatures. In the first case, the coating was performed on the substrates that were present at room temperature (RT), and then the substrates were heated at 250 °C on a heating plate in the second case. This temperature was chosen because spray pyrolysis decomposition does not occur below 200 °C, and at temperatures above 300 °C, homogeneous, compact films are typically formed. Furthermore, at this temperature range, porosity can be improved during post-annealing treatment by some decrease in film mass due to the release of some oxygen and water still present in the film after deposition onto the substrate at Ts < 300 °C [39]. The coatings were also repeated from two different gun to substrate distances, 20 cm, and 30 cm. Additionally, different volumes, 1 mL and 2 mL, were used to investigate the effect of the volume on the prepared thin film properties. For all the depositions, a 6 kPa spray pressure was maintained. The samples were annealed in a muffle furnace at 450 °C for 2 h. The names of the samples are presented in Table 1.

To prepare the dye solution, 1 mg of Rhodamine B was taken in 1 L of deionized water. Coated ZnO samples were added in 50 mL of dye solution to study their effect on dye photodegradation. The adsorption–desorption equilibrium of the reaction mixture was attained before exposure to UV light by keeping the mixture in the dark for 1 h. To evaluate the photoactivity of the prepared ZnO coatings, the photodegradation of an organic pollutant rhodamine B was tested under UV irradiation. ZnO has maximum absorbance in the UV region, so photodegradation was observed in a UV photocatalytic reactor at room temperature. The 15 W UV-A lights that have a wavelength range of 320 nm to a visible light cut-off at 380 nm were employed for the photodegradation. The lamp’s peak wavelength is 366 nm.

The size distribution and the phase of thin films were measured via scanning electron microscopy (SEM, JOEL JSM-6490 A) and X-ray diffraction (XRD, Stoe D-64295 Darmstadt), respectively. For XRD, a Cu K_a_ source was used for X-ray generation. The results showed better size distribution of nanoparticles for heated thin films. The surface roughness was measured by atomic force microscopy (AFM, JEOL-SPM 5200), which was approximately 55 nm. To measure the absorbance of thin films, steady-state UV–Vis spectroscopy (Jenway 7315 UV–Vis spectrometer) was performed, and the results showed that all the samples were active in the UV region and transparent in the visible region (from 500–800 nm). Contact angle measurements were conducted for all the samples to test the wettability of the surfaces using contact angle goniometer (Drop shape analyzer-DSA25, Kruss), which had a measuring range from 0 < θ < 180°. The measurements were performed using automatic analysis using two base points. The ZnO films switched their hydrophilic nature to hydrophobic when they were deposited on the heated substrates. Furthermore, the photoactivity of the thin films was measured by photodegradation of Rhodamine B (RhB) dye. The oxygen vacancies found in ZnO TFs help in degrading the organic structure of the dye molecules [40]. Rhodamine B (RhB) is an organic dye. According to Beer–Lambert law, the concentration of the organic dye (RhB) in the solution is proportional to its absorption. The following equations can be used to compute the degradation efficiency.

Degradation Efficiency = [(C_0_ − C)/C_0_] × 100%

where C_0_ is initial concentration (at t = 0) and C is the concentration of the dye after 10 h reaction time.

The photocatalysis reaction rate (k_app_) can be calculated using the following first-order reaction equation.

ln(C/C_0_) = −kKt = k_app_t

where K is the adsorption constants, k is the first-order reaction rate constant, and t is the irradiation time. The apparent first-order rate constant, k_app_, can be calculated using the best-fit straight line of the semi-logarithmic ln(C/C_0_) vs. t plot [41].

## 3. Results and Discussion

**Figure 2:**
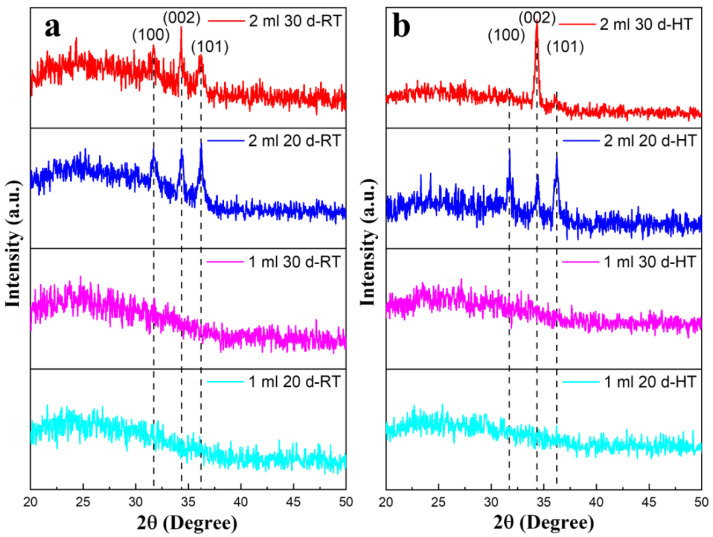
The XRD results of all the ZnO thin films prepared at (**a**) room temperature and (**b**) the heated substrates.

The crystallographic properties of all the samples determined using XRD are given in Figure 2. Figure 2a shows the results for the samples that were prepared at room temperature (RT), and Figure 2b illustrates the samples when the substrate was heated at 250 °C. It was observed that all the samples, deposited on substrates at RT or heated, with 1 mL solution showed an amorphous nature. However, for all the samples deposited with 2 mL solution, crystallinity was observed. Kim et al. stated that below a certain film thickness, the grown thin films show an amorphous character [42], which can be explained by the fact that in the case of the 2 mL samples, there is more ZnO coating, which leads to an arrangement of the structure and a variation in the crystallographic attributes [43]. For the RT samples, better crystallinity was observed when the distance for the solution deposition was 20 cm. However, better crystallinity and texture were observed in the case of the sample deposited on the heated substrate with 30 cm, while the induction of crystallinity could still be observed with the sample deposited at 20 cm. For all the crystalline thin films, the hexagonal wurtzite crystallographic structure of ZnO was observed. (100), (002), and (101) planes were determined by the major peaks that were consistent with the literature (JCPDS 36-1451). For the heated samples deposited at a 30 cm distance, the stronger relative intensity of the (002) peak confirmed the ZnO preferable orientation, the c-axis, which is the kinetically preferred orientation in ZnO TFs [38].

**Figure 3:**
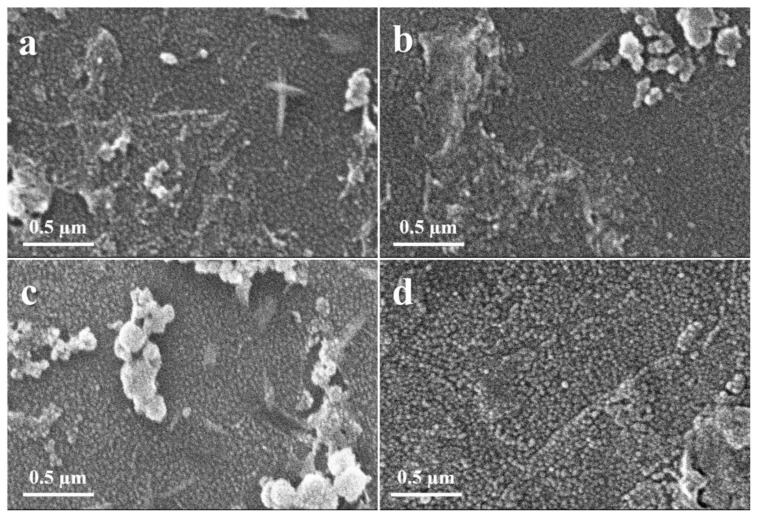
The SEM images of the ZnO coatings deposited at room temperature were prepared using (**a**,**b**) 1 mL solution with 20 cm and 30 cm distances and (**c**,**d**) using 2 mL solution with 20 cm and 30 cm distances.

**Figure 4:**
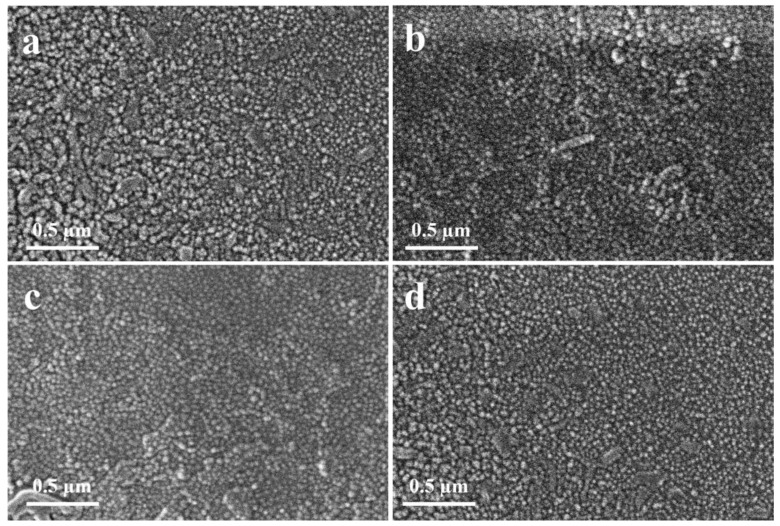
The SEM images of the ZnO coatings deposited at heated substrates at 250 °C were prepared using (**a**,**b**) 1 mL solution with 20 cm and 30 cm distances and (**c**,**d**) using 2 mL solution with 20 cm and 30 cm distances.

Figure 3 and Figure 4 show the morphology and uniformity of the coatings that were prepared using the SP, which were obtained by SEM. Figure 3 shows the coatings of the ZnO that were prepared at room temperature. It can be observed that with 1 mL solution, the nanoparticles are not that defined, and the structures are quite agglomerated irrespective of the distance of the spray gun to the substrate, which is shown in Figure 3a,b. However, when the 2 mL solution is used, the nanoparticles have more defined structures even when the spray distance is 30 cm, and the size of the nanoparticles (NPs) range from 40 nm to 50 nm. The NPs are quite uniform in size and very little agglomeration can be observed. The coatings prepared with heated substrates at 250 °C are shown in Figure 4. For the samples prepared with 1 mL solution, the results are shown in Figure 4a,b. In the case of the coatings that were carried out at a 20 cm distance, the nanoparticles are large, in the range of size from 50 nm to 150 nm, and the particle size distribution is not uniform. However, the sample prepared using a 30 cm spray distance has a uniform size distribution of the NPs, with 30 nm to 50 nm size. In the case of samples prepared using the 2 mL solution, the results are shown in Figure 4c,d. The samples that were prepared using a 30 cm spray distance show a uniform size distribution for the NPs and the size of the NPs range from 20 nm to 35 nm. For the samples that were prepared using a 20 cm spray distance, the grain size of the NPs increases, while they range from 40 nm to 100 nm, and the size distribution is broad.

**Figure 5:**
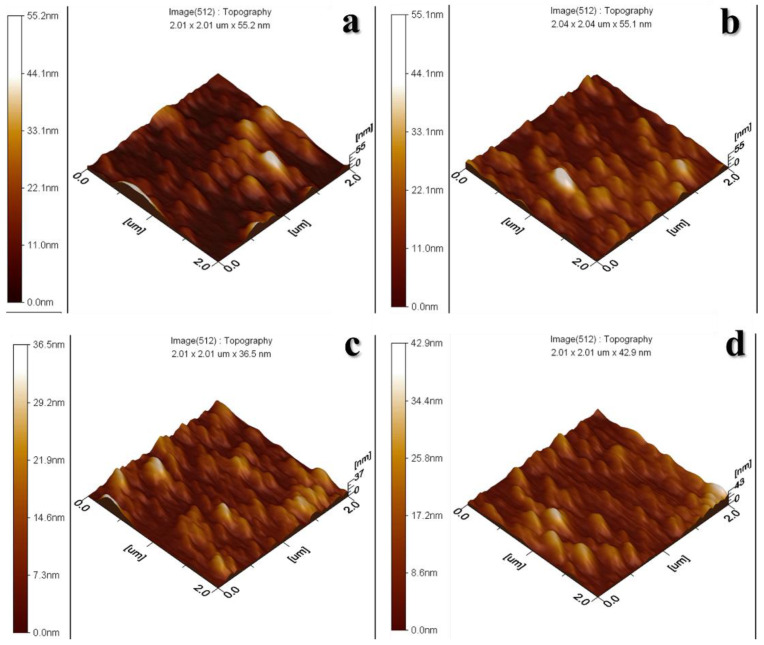
The AFM images of the substrates were prepared using 2 mL solution, (**a**,**b**) were deposited with 20 cm and 30 cm spray distances at room temperature, and (**c**,**d**) were deposited with 20 cm and 30 cm spray distances at heated substrates.

The surface roughness of the prepared ZnO TFs was determined using AFM. The images were taken on 2 × 2 µm^2^. All the samples were compared based on the varying aforementioned parameters. The images for the samples deposited with 1 mL solution are presented in Figure S1 in the Supplementary Materials. For the samples deposited with 2 mL solution, the results are presented in Figure 5. Figure S1 in the Supplementary Materials and Figure 5 show the surface topology. All these samples show better surface coverage and roughness as compared to the samples deposited with 1 mL solution. However, the samples that are deposited at a distance of 20 cm to the substrate show the highest roughness. Hence, it can be observed that increasing the volume of the solution and decreasing the distance of solution deposition results in films with high roughness. Other than the surface topology, the roughness of the surface was also studied, which can be described by the root mean square average of the height deviations on the bulging surface. The values of the root mean square (R_q_) for all the samples are given in Table 2.

**Figure 6:**
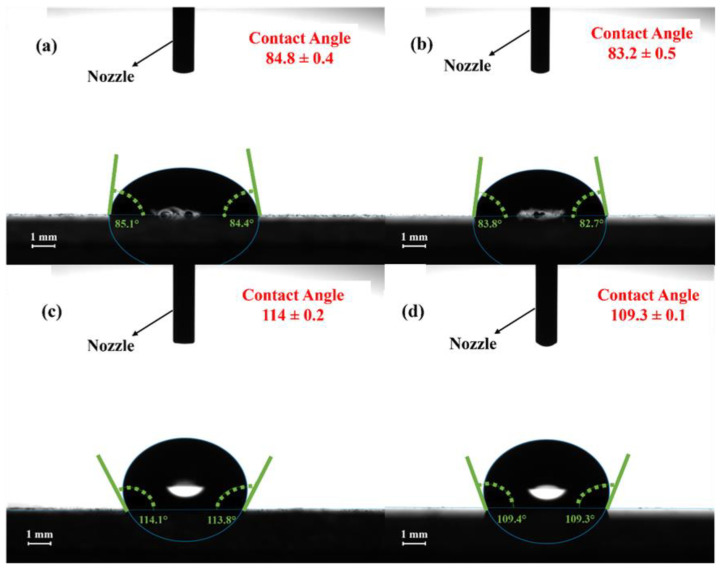
The contact angle images of the substrates prepared with 2 mL solution (**a**,**b**) deposited with 20 cm and 30 cm spray distances at room temperature and (**c**,**d**) deposited with 20 cm and 30 cm spray distances at heated substrates.

The contact angle measurement was made to evaluate the wetting property of the prepared samples. The apparent contact angle decreases as the solid surface roughness increases and the hydrophilicity improves when the solid surface is hydrophilic (contact angle < 90°). The apparent contact angle will increase with the solid surface roughness if the solid surface is hydrophobic (contact angle > 90°) [44]. The images for the samples deposited using 1 mL solution for both the RT and the heated samples are shown in Figure S2 in the Supplementary Materials. For the samples deposited with the 2 mL solution, it can be observed that when they are deposited at room temperature by varying the distance and being further annealed, they show a slight hydrophilic character and a contact angle of ≤90°. The contact angles images of the samples deposited at RT and that are further annealed are shown in Figure 6a,b. However, when the samples are deposited on heated substrates by changing the distance and being further annealed, the films start to show hydrophobic character with a contact angle of ≥103°. Moreover, it can also be observed that when the volume of the spray is increased in the case of 2 mL samples, the contact angle also increases compared to the samples prepared with 1 mL solution. Additionally, by changing the distance, it is then observed that the contact angle is less for the samples prepared from a 30 cm distance with the same volume of a solution, which is shown in Figure 6b,d, compared to the samples prepared from a 20 cm distance, which are shown in Figure 6a,c. The water contact angle of the substrates only reflects their surface qualities; however, their water removal capacity is heavily influenced by their pore structure and surface wettability [45]. The contact angle changes as a result of increased surface roughness as indicated in the AFM results. Moreover, samples prepared by maintaining a distance of 30 cm from the gun to the substrate have uniform particle size distribution. The contact angle measurements are given in Table 3.

This change in the contact angle can be demonstrated, as observed in titania thin films [46], the surface energy decreases with a higher degree of crystallinity. In the current study, both hydrophilic and hydrophobic sites are present in the RT samples due to the presence of amorphous and crystalline phases on the surface. These phases can be confirmed from the XRD data in Figure 2a. In the case of HT samples, Figure 2b shows that the crystallinity of the HT thin films is increased (increase in the intensity of hkl peaks and decrease in the broad background). Due to the increase in the crystallinity of HT thin films the surface energy decreases, and the contact angle increases. This is in addition to the larger particles present on the surface and surface roughness of the RT thin films as compared to that of HT thin films, which can be observed from SEM micrographs (Figure 3 and Figure 4) and AFM (Figure 5), respectively.

**Figure 7:**
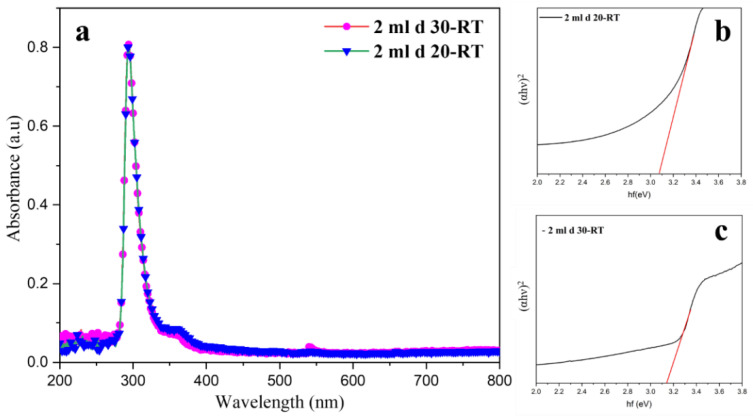
(**a**) UV–Vis of RT samples, (**b**,**c**) bandgap of the RT samples.

UV–Vis spectroscopy was performed to obtain the absorbance spectra of the prepared ZnO TFs. The results of the bandgap for all the heated samples are shown in Figure S3 in the Supplementary Materials. The band gaps of the RT-prepared samples are shown in Figure 7. It can be observed that when the substrates are heated at 250 °C, the bandgap increases. When the solution is sprayed from a distance far from the substrate, less solution is deposited. The bandgap of prepared samples ranges from 3.21 eV to 3.5 eV. The spectra show that the ZnO TFs are highly active in the UV region while being completely transparent in the visible region.

**Figure 8:**
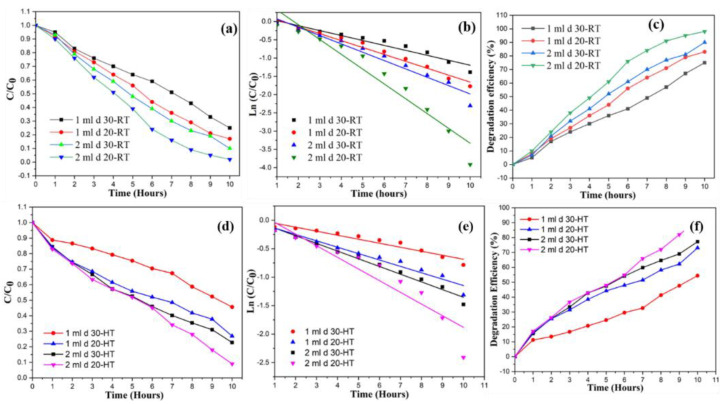
(**a**,**d**) Photodegradation at RT and heated substrate, (**b**,**e**) determination of the rate of reactions at RT and heated substrate, (**c**,**f**) degradation efficiency of RT and heated substrate.

A small bandgap is necessary for a photocatalyst to demonstrate activity. Another important requirement for organic compound degradation is if the redox potential of the H_2_O/OH couple is inside the range of the semiconductor’s bandgap [14]. The progress of the photodegradation was studied using Beer–Lambert law (*A* = *εcl*) with a UV–Vis spectrophotometer [47]. The photodegradation was measured after every one-hour interval. It can be observed that irrespective of the deposition temperature used, such as the RT samples or the heated samples, the samples with the 2 mL volume of solution sprayed from a 20 cm distance have the best degradation efficiency compared to the samples prepared with the 1 mL solution, which is shown in Figure 8. This is because more ZnO NPs are formed on the substrate in these samples due to the shorter distance. Moreover, as observed from SEM micrographs in Figure 3 and Figure 4, the size of the NPs is smaller in heated substrates as compared to the RT ones, which increases the surface area for the photocatalytic reactions. The increase in the surface roughness of these samples is evident from the AFM images provided in Figure 5, and this also contributes to the enhanced photoactivity of the thin films. Owing to the increased surface area of the NPs, more active sites are available for the dye molecules. This enhances the photoactivity of ZnO TFs.

**Figure 9:**
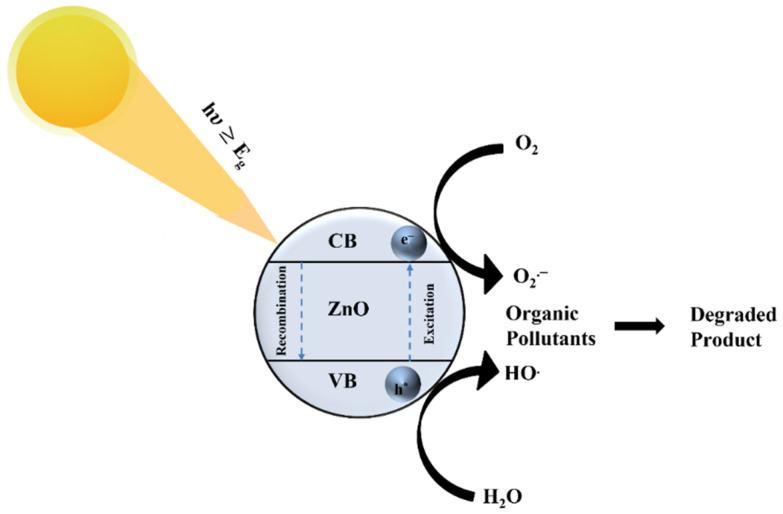
Mechanism of photodegradation by the ZnO thin films under UV light irradiation.

Based on photoactivity analysis, a plausible photo-response mechanism is proposed [48]. Under UV light irradiation, the electron in the ZnO valance band is transferred to a conduction band because the corresponding energy is higher than its bandgap. The photogenerated holes can (a) directly oxidize the dye, (b) react with the H_2_O molecules, or (c) form hydroxyl radicals (•OH) with the OH^−^ ions. Additionally, the electrons on the catalyst surface reduce the oxygen molecules to form superoxide radicals (•O_2_^−^). Therefore, the dye molecules are degraded by the generation of •OH and •O_2_^−^. As the band gap of ZnO is 3.3 eV, it is active under UV light irradiation. The plausible photo-response mechanism is that under UV light, photogenerated electron-hole pairs are generated in the photocatalyst. The electrons (e^−^) go to the conduction band while holes (h^+^) remain in the valence band. The photogenerated holes could either directly oxidize adsorbed dyes on the surface of ZnO or react with hydroxyl (OH^−^) or H_2_O molecules to generate hydroxyl radicals (•OH). The photoinduced electrons reduce oxygen (O_2_) adsorbed on the photocatalyst surface into superoxide radical (•O_2_^−^). Finally, the dyes are decomposed by the generated •OH and •O_2_^−^. This mechanism is shown in Figure 9 and is given as follows.

ZnO + hv → h^+^ + e^−^

h^+^ + OH^−^/H_2_O → •OH

e^−^ + O_2_ → •O_2_^−^

•OH and •O_2_^−^ + dye molecules → CO_2_ + H_2_O + inorganic acids

## 4. Conclusions

In this study, we optimized ZnO TFs prepared via SPT on substrates present at room temperature and heated substrates at 250 °C for exploiting wettability. The properties of the films were tailored via varying different parameters, such as by changing the temperature of the substrates, the distance of the spray gun to the substrate, and the volume of the solution used. The samples prepared with a 2 mL solution show better surface coverage of the substrates with a uniform particle size distribution, which can be seen by the SEM images. The AFM results show that the surface roughness increases when the distance of the spray is maintained at 20 cm for all samples either deposited at room temperature or on heated substrates. However, in the case of heated substrates, the surface roughness is slightly reduced as clearly indicated by the R_q_ values in Table 2. Intrinsically, ZnO is hydrophilic. However, when the 2 mL solution is used for the deposition on heated substrates, the wettability of the films increases, and they start to show a hydrophobic character. The samples showing hydrophilic characteristics at RT started showing hydrophobic characteristics when the same was deposited at a heated substrate. The XRD results showed higher degree of crystallinity for samples deposited on heated substrates. Because of the higher crystallinity the surface energy decreases, and the contact angle is increased. The photoactivity of the samples is tested by the photodegradation of the rhodamine B dye under UV light irradiation. It was observed that when the substrate is deposited with the 2 mL volume of solution sprayed from a 20 cm distance, the samples provided the best degradation efficiency. Moreover, it can be concluded that when the substrates are heated, they give a pronounced coating with enhanced self-cleaning properties and better photoactivity. Additionally, these thin films can be employed over windows to block UV rays without compromising the daylight as they are transparent in the visible region. Additionally, this technique is cost-effective and efficient so can be easily adopted for industrial purposes.

## Figures and Tables

**Table 1:** Samples’ names that were prepared by varying parameters such as temperature, the distance of the spray gun to the substrate, and volume of the solution.

Sample Name (ZnO)	Temperature	Distance	Volume
1 mL 20 d-RT	Room Temperature	20 cm	1 mL
1 mL 20 d-HT	250 °C	20 cm	1 mL
1 mL 30 d-RT	Room Temperature	30 cm	1 mL
1 mL 30 d-HT	250 °C	30 cm	1 mL
2 mL 20 d-RT	Room Temperature	20 cm	2 mL
2 mL 20 d-HT	250 °C	20 cm	2 mL
2 mL 30 d-RT	Room Temperature	30 cm	2 mL
2 mL 30 d-HT	250 °C	30 cm	2 mL

**Table 2:** Surface roughness values of all the prepared samples.

Samples Name (ZnO)	R_q_ (nm)
1 mL 20 d-RT	9.52 ± 0.97
1 mL 20 d-HT	6.90 ± 0.72
1 mL 30 d-RT	8.00 ± 0.71
1 mL 30 d-HT	7.58 ± 0.68
2 mL 20 d-RT	9.44 ± 1.08
2 mL 20 d-HT	6.68 ± 0.62
2 mL 30 d-RT	8.47 ± 0.90
2 mL 30 d-HT	7.25 ± 0.74

**Table 3:** Contact angle of all the prepared samples.

Sample Name (ZnO)	Contact Angle
1 mL 20 d-RT	74.5°
1 mL 20 d-HT	108°
1 mL 30 d-RT	67°
1 mL 30 d-HT	102°
2 mL 20 d-RT	85°
2 mL 20 d-HT	114°
2 mL 30 d-RT	83°
2 mL 30 d-HT	109°

## Data Availability

The data is available on reasonable request.

## Supplementary Materials

The following supporting information can be downloaded at: https://www.mdpi.com/article/10.3390/ma15093364/s1, Figure S1: AFM images of substrates prepared with 1 mL sol (a,b) deposited with 20 and 30 cm spray distance at room temperature and (c,d) deposited with 20 and 30 cm spray distance at pre-heated substrates; Figure S2: Contact angle images of substrates prepared with 1 mL sol (a,b) deposited with 20 and 30 cm spray distance at room temperature and (c,d) deposited with 20 and 30 cm spray distance at pre-heated substrates; Figure S3: Bandgap of heated ZnO samples.

## References

[1] Ganga G.L., Nastasi F., Campagna S., Puntoriero F. (2009). Photoinduced Water Oxidation Sensitized by a Tetranuclear Ru(II) Dendrimer. Dalton Trans..

[2] Parkin I.P., Palgrave R.G. (2005). Self-Cleaning Coatings. J. Mater. Chem..

[3] Ragesh P., Ganesh V.A., Nair S.V., Nair A.S. (2014). A Review on ‘Self-Cleaning and Multifunctional Materials’. J. Mater. Chem. A.

[4] Gao N., Yan Y. (2012). Characterisation of Surface Wettability Based on Nanoparticles. Nanoscale.

[5] Midtdal K., Jelle B.P. (2013). Self-Cleaning Glazing Products: A State-of-the-Art Review and Future Research Pathways. Sol. Energy Mater. Sol. Cells.

[6] Liang Z., Zhao L., Meng W., Zhong C., Wei S., Dong B., Xu Z., Wan L., Wang S. (2017). Tungsten-Doped Vanadium Dioxide Thin Films as Smart Windows with Self-Cleaning and Energy-Saving Functions. J. Alloys Compd..

[7] Kafizas A., Kellici S., Darr J.A., Parkin I.P. (2009). Titanium Dioxide and Composite Metal/Metal Oxide Titania Thin Films on Glass: A Comparative Study of Photocatalytic Activity. J. Photochem. Photobiol. A Chem..

[8] Baudys M., Krýsa J., Mills A. (2017). Smart Inks as Photocatalytic Activity Indicators of Self-Cleaning Paints. Catal. Today.

[9] Graziani L., Quagliarini E., Bondioli F., D’Orazio M. (2014). Durability of Self-Cleaning TiO_2_ Coatings on Fired Clay Brick Façades: Effects of UV Exposure and Wet & Dry Cycles. Build. Environ..

[10] Erbil H.Y., Demirel A.L., Avcı Y., Mert O. (2003). Transformation of a Simple Plastic into a Superhydrophobic Surface. Science.

[11] Sakai N., Fujishima A., Watanabe T., Hashimoto K. (2003). Quantitative Evaluation of the Photoinduced Hydrophilic Conversion Properties of TiO_2_ Thin Film Surfaces by the Reciprocal of Contact Angle. J. Phys. Chem. B.

[12] Sutha S., Suresh S., Raj B., Ravi K.R. (2017). Transparent Alumina Based Superhydrophobic Self–Cleaning Coatings for Solar Cell Cover Glass Applications. Sol. Energy Mater. Sol. Cells.

[13] Diby N.D., Wang J., Duan Y. (2020). Motion Behaviour of Water-Droplet on Alternate Superhydrophobic/Hydrophilic ZnO Wetting-Patterned Surface. Surf. Eng..

[14] Soltani N., Saion E., Hussein M.Z., Erfani M., Abedini A., Bahmanrokh G., Navasery M., Vaziri P. (2012). Visible Light-Induced Degradation of Methylene Blue in the Presence of Photocatalytic ZnS and CdS Nanoparticles. Int. J. Mol. Sci..

[15] Mote V.D., Dargad J.S., Dole B.N. (2013). Effect of Mn Doping Concentration on Structural, Morphological and Optical Studies of ZnO Nano-Particles. Nanosci. Nanoeng..

[16] Intarasuwan K., Amornpitoksuk P., Suwanboon S., Graidist P. (2017). Photocatalytic Dye Degradation by ZnO Nanoparticles Prepared from X_2_C_2_O_4_ (X = H, Na and NH_4_) and the Cytotoxicity of the Treated Dye Solutions. Sep. Purif. Technol..

[17] Badawy M.I., Mahmoud F.A., Abdel-Khalek A.A., Gad-Allah T.A., Samad A.A.A. (2014). Solar Photocatalytic Activity of Sol–Gel Prepared Ag-Doped ZnO Thin Films. Desalin. Water Treat..

[18] Wang J., Li Y., Kong Y., Zhou J., Wu J., Wu X., Qin W., Jiao Z., Jiang L. (2015). Non-Fluorinated Superhydrophobic and Micro/Nano Hierarchical Al Doped ZnO Film: The Effect of Al Doping on Morphological and Hydrophobic Properties. RSC Adv..

[19] Sun J.-H., Dong S.-Y., Wang Y.-K., Sun S.-P. (2009). Preparation and Photocatalytic Property of a Novel Dumbbell-Shaped ZnO Microcrystal Photocatalyst. J. Hazard. Mater..

[20] Xie W., Li Y., Sun W., Huang J., Xie H., Zhao X. (2010). Surface Modification of ZnO with Ag Improves Its Photocatalytic Efficiency and Photostability. J. Photochem. Photobiol. A Chem..

[21] Trandafilović L.V., Jovanović D.J., Zhang X., Ptasińska S., Dramićanin M.D. (2017). Enhanced Photocatalytic Degradation of Methylene Blue and Methyl Orange by ZnO:Eu Nanoparticles. Appl. Catal. B Environ..

[22] Rahman A., Jayaganthan R., Jain R.K., Chawla A.K., Chandra R., Ambardar R. (2013). Study of Nanostructured Al Doped ZnO Films. Surf. Eng..

[23] Akram M.A., Javed S., Mujahid M. (2015). Synthesis and Surface Modification of ZnO Nanorods Arrays. Adv. Mater. Res..

[24] Akram M.A., Javed S., Xu J., Mujahid M., Lee C.-S. (2015). Arrays of ZnO/CuIn_x_Ga_1−x_Se_2_ Nanocables with Tunable Shell Composition for Efficient Photovoltaics. J. Appl. Phys..

[25] Aftab Akram M., Javed S., Islam M., Mujahid M., Safdar A. (2016). Arrays of CZTS Sensitized ZnO/ZnS and ZnO/ZnSe Core/Shell Nanorods for Liquid Junction Nanowire Solar Cells. Sol. Energy Mater. Sol. Cells.

[26] Stieberova B., Zilka M., Ticha M., Freiberg F., Caramazana-González P., McKechnie J., Lester E. (2017). Application of ZnO Nanoparticles in a Self-Cleaning Coating on a Metal Panel: An Assessment of Environmental Benefits. ACS Sustain. Chem. Eng..

[27] Kenanakis G., Giannakoudakis Z., Vernardou D., Savvakis C., Katsarakis N. (2010). Photocatalytic Degradation of Stearic Acid by ZnO Thin Films and Nanostructures Deposited by Different Chemical Routes. Catal. Today.

[28] Farahani N., Kelly P.J., West G., Ratova M., Hill C., Vishnyakov V. (2011). Photocatalytic Activity of Reactively Sputtered and Directly Sputtered Titania Coatings. Thin Solid Film..

[29] Dong B., Yu X., Dong Z., Yang X., Wu Y. (2017). Facile Synthesis of ZnO Nanoparticles for the Photocatalytic Degradation of Methylene Blue. J. Sol-Gel Sci. Technol..

[30] Ravichandran K., Saravanakumar K., Muruganantham G., Sakthivel B. (2010). Low Temperature Fabrication of Highly Transparent Conducting SnO_2_–ZnO Films by Inexpensive, Simplified Spray Technique. Surf. Eng..

[31] Navidpour A.H., Hosseinzadeh A., Zhou J.L., Huang Z. (2021). Progress in the Application of Surface Engineering Methods in Immobilizing TiO_2_ and ZnO Coatings for Environmental Photocatalysis. Catal. Rev. Sci. Eng..

[32] Hunge Y.M., Yadav A.A., Kulkarni S.B., Mathe V.L. (2018). A Multifunctional ZnO Thin Film Based Devices for Photoelectrocatalytic Degradation of Terephthalic Acid and CO_2_ Gas Sensing Applications. Sens. Actuators B Chem..

[33] Tarwal N.L., Patil P.S. (2010). Superhydrophobic and Transparent ZnO Thin Films Synthesized by Spray Pyrolysis Technique. Appl. Surf. Sci..

[34] Ravichandran K., Sindhuja E., Uma R., Arun T. (2017). Photocatalytic Efficacy of ZnO Films—Light Intensity and Thickness Effects. Surf. Eng..

[35] Antar B., Youcef B. (2020). Facile Synthesis of Spray Pyrolyzed ZnO/NiO Nanocomposites Thin Films. Phosphorus Sulfur Silicon Relat. Elem..

[36] Islam M.R., Azam M.G. (2021). Enhanced Photocatalytic Activity of Mg-Doped ZnO Thin Films Prepared by Sol–Gel Method. Surf. Eng..

[37] Zirak M., Alehdaghi H., Shakoori A.M. (2021). Preparation of ZnO-Carbon Quantum Dot Composite Thin Films with Superhydrophilic Surface. Mater. Technol..

[38] Salam S., Islam M., Akram A. (2013). Sol–Gel Synthesis of Intrinsic and Aluminum-Doped Zinc Oxide Thin Films as Transparent Conducting Oxides for Thin Film Solar Cells. Thin Solid Film..

[39] Allah F.K., Abé S.Y., Núñez C.M., Khelil A., Cattin L., Morsli M., Bernède J.C., Bougrine A., del Valle M.A., Díaz F.R. (2007). Characterisation of Porous Doped ZnO Thin Films Deposited by Spray Pyrolysis Technique. Appl. Surf. Sci..

[40] Kalyanasundaram K. (2013). Photochemical Applications of Solar Energy: Photocatalysis and Photodecomposition of Water. Photochemistry.

[41] Dodoo-Arhin D., Asiedu T., Agyei-Tuffour B., Nyankson E., Obada D., Mwabora J.M. (2021). Photocatalytic Degradation of Rhodamine Dyes Using Zinc Oxide Nanoparticles. Mater. Today Proc..

[42] Kim S.K., Hoffmann-Eifert S., Reiners M., Waser R. (2010). Relation Between Enhancement in Growth and Thickness-Dependent Crystallization in ALD TiO_2_ Thin Films. J. Electrochem. Soc..

[43] Kozlovskiy A., Shlimas I., Dukenbayev K., Zdorovets M. (2019). Structure and Corrosion Properties of Thin TiO_2_ Films Obtained by Magnetron Sputtering. Vacuum.

[44] Li C., Zhang J., Han J., Yao B. (2021). A Numerical Solution to the Effects of Surface Roughness on Water–Coal Contact Angle. Sci. Rep..

[45] Wang X.L., Wang W.K., Qu Z.G., Ren G.F., Wang H.C. (2021). Surface Roughness Dominated Wettability of Carbon Fiber in Gas Diffusion Layer Materials Revealed by Molecular Dynamics Simulations. Int. J. Hydrogen Energy.

[46] Shirolkar M.M., Phase D., Sathe V., Rodríguez-Carvajal J., Choudhary R.J., Kulkarni S.K. (2011). Relation between Crystallinity and Chemical Nature of Surface on Wettability: A Study on Pulsed Laser Deposited TiO_2_ Thin Films. J. Appl. Phys..

[47] Khan R., Riaz A., Rabeel M., Javed S., Jan R., Akram M.A. (2019). TiO_2_@NbSe_2_ Decorated Nanocomposites for Efficient Visible-Light Photocatalysis. Appl. Nanosci..

[48] Riaz A., Ashraf A., Taimoor H., Javed S., Akram M.A., Islam M., Mujahid M., Ahmad I., Saeed K. (2019). Photocatalytic and Photostability Behavior of Ag- and/or Al-Doped ZnO Films in Methylene Blue and Rhodamine B under UV-C Irradiation. Coatings.

