# Supplementary material for: Controlling the Wettability of ZnO Thin Films by Spray Pyrolysis for Photocatalytic Applications
Source: Materials (Basel). 2022 May 7;15(9):3364. doi: 10.3390/ma15093364 (PMC9105918; doi:10.3390/ma15093364)
Supplement: Supplementary file 1 [file materials-15-03364-s001.zip › materials-1681453-supplementary.pdf]

materials

MDPI

Article

Controlling the Wettability of ZnO Thin Films by Spray Pyrolysis for Photocatalytic Applications

Muhammad Rabeel [1,2], Sofia Javed [1,*], Ramsha Khan [1], Muhammad Aftab Akram [1], Shania Rehman [2], Deok-kee Kim [2,*] and Muhammad Farooq Khan [2,*]

[1] Nano Synthesis Laboratory, School of Chemical and Materials Engineering, National University of Sciences and Technology, H-12, Islamabad 44000, Pakistan; rana.rabeel12@gmail.com (M.R.); khanramsha94@gmail.com (R.K.); aftabakram@scme.nust.edu.pk (M.A.A.)

[2] Department of Electrical Engineering, Sejong University, 209 Neungdong-ro, Gwangjin-gu, Seoul 05006, Korea; shania.rehman19@gmail.com

* Correspondence: sofia.javed@scme.nust.edu.pk (S.J.); deokkeekim@sejong.ac.kr (D.-k.K.); mfk@sejong.ac.kr (M.F.K.)

Abstract: Herein, we synthesized the zinc oxide (ZnO) thin films (TFs) deposited on glass substrates via spray pyrolysis (SP) to prepare self-cleaning glass. Various process parameters were used to optimize photocatalytic performance. Substrates were coated at room temperature (RT) and 250 °C with a 1 mL or 2 mL ZnO solution while maintaining a distance from the spray gun to the substrate of 20 cm or 30 cm. Several characterization techniques, i.e., XRD, SEM, AFM, and UV–Vis were used to determine the structural, morphological, and optical characteristics of the prepared samples. The wettability of the samples was evaluated using contact angle measurements. As ZnO is hydrophilic in nature, the RT deposited samples showed a hydrophilic character, whereas the ZnO TFs deposited at 250 °C demonstrated a hydrophobic character. The XRD results showed a higher degree of crystallinity for samples deposited on heated substrates. Because of this higher crystallinity, the surface energy decreased, and the contact angle increased. Moreover, by using 2 mL solution, better surface coverage and roughness were obtained for the ZnO TFs. However, by exploiting the distance of the spray to the samples size distribution and surface coverage can be controlled, the samples deposited at 30 mL showed a uniform particle size distribution from 30–40 nm. In addition, the photoactivity of the samples was tested by the degradation of rhodamine B dye. Substrates prepared with a 2 mL solution sprayed at 20 cm showed higher dye degradation than other samples, which can play a vital role in self-cleaning. Hence, by changing the said parameters, the ZnO thin film properties on glass substrates were optimized for self-cleaning diversity.

Keywords: zinc oxide thin films; spray pyrolysis; contact angle; photocatalysis; rhodamine B; wettability

Citation: Rabeel, M.; Javed, S.; Khan, R.; Akram, M.A.; Rehman, S.; Kim, D.-k.; Khan, M.F. Controlling the Wettability of ZnO Thin Films by Spray Pyrolysis for Photocatalytic Applications. *Materials* **2022**, *15*, 3364. https://doi.org/10.3390/ma15093364

Received: 30 March 2022
Accepted: 4 May 2022
Published: 7 May 2022

Publisher's Note: MDPI stays neutral with regard to jurisdictional claims in published maps and institutional affiliations.

Copyright: © 2022 by the authors. Licensee MDPI, Basel, Switzerland. This article is an open access article distributed under the terms and conditions of the Creative Commons Attribution (CC BY) license (https://creativecommons.org/licenses/by/4.0/).

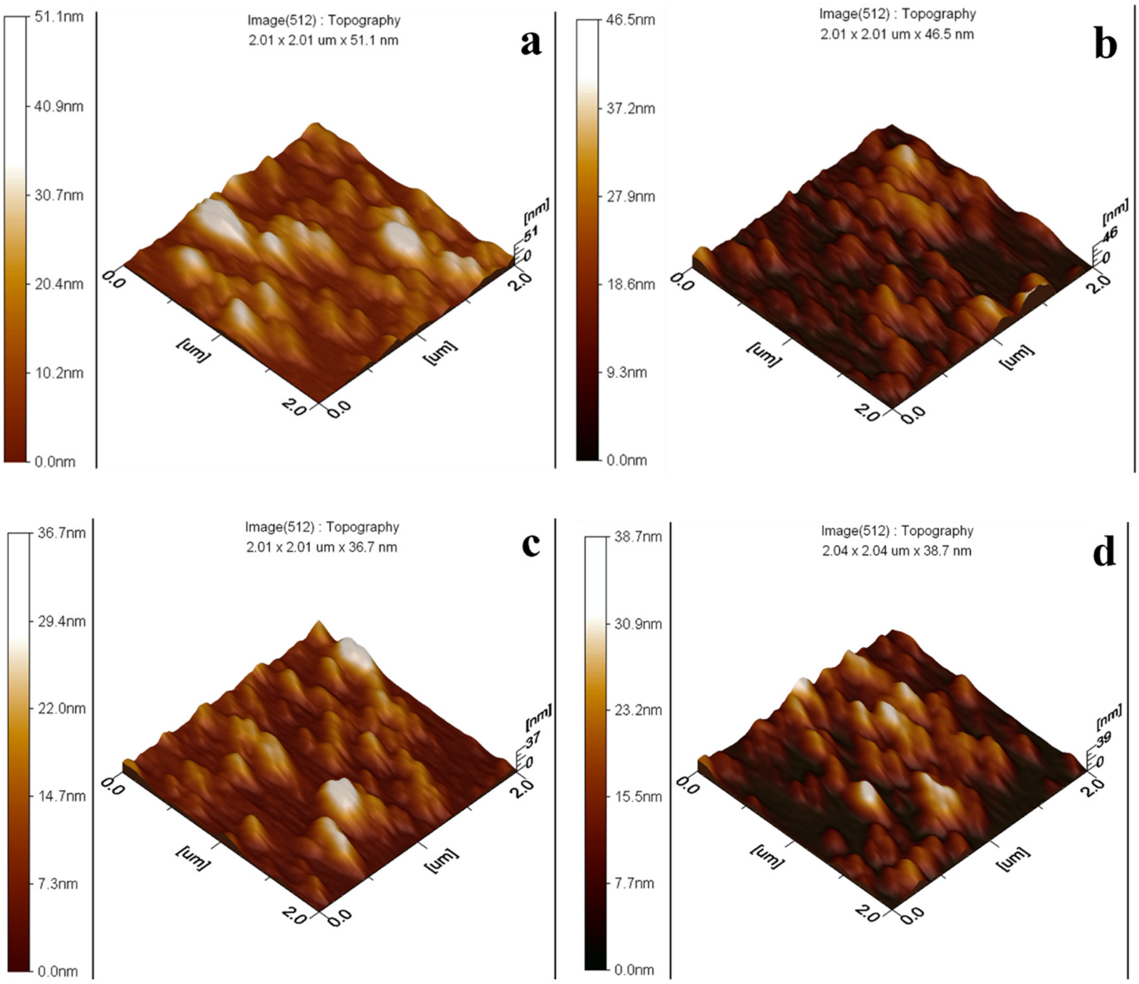

Figure S1. AFM images of substrates prepared with 1 ml sol (a, b) deposited with 20 and 30 cm spray distance at room temperature and (c, d) deposited with 20 and 30 cm spray distance at pre-heated substrates.

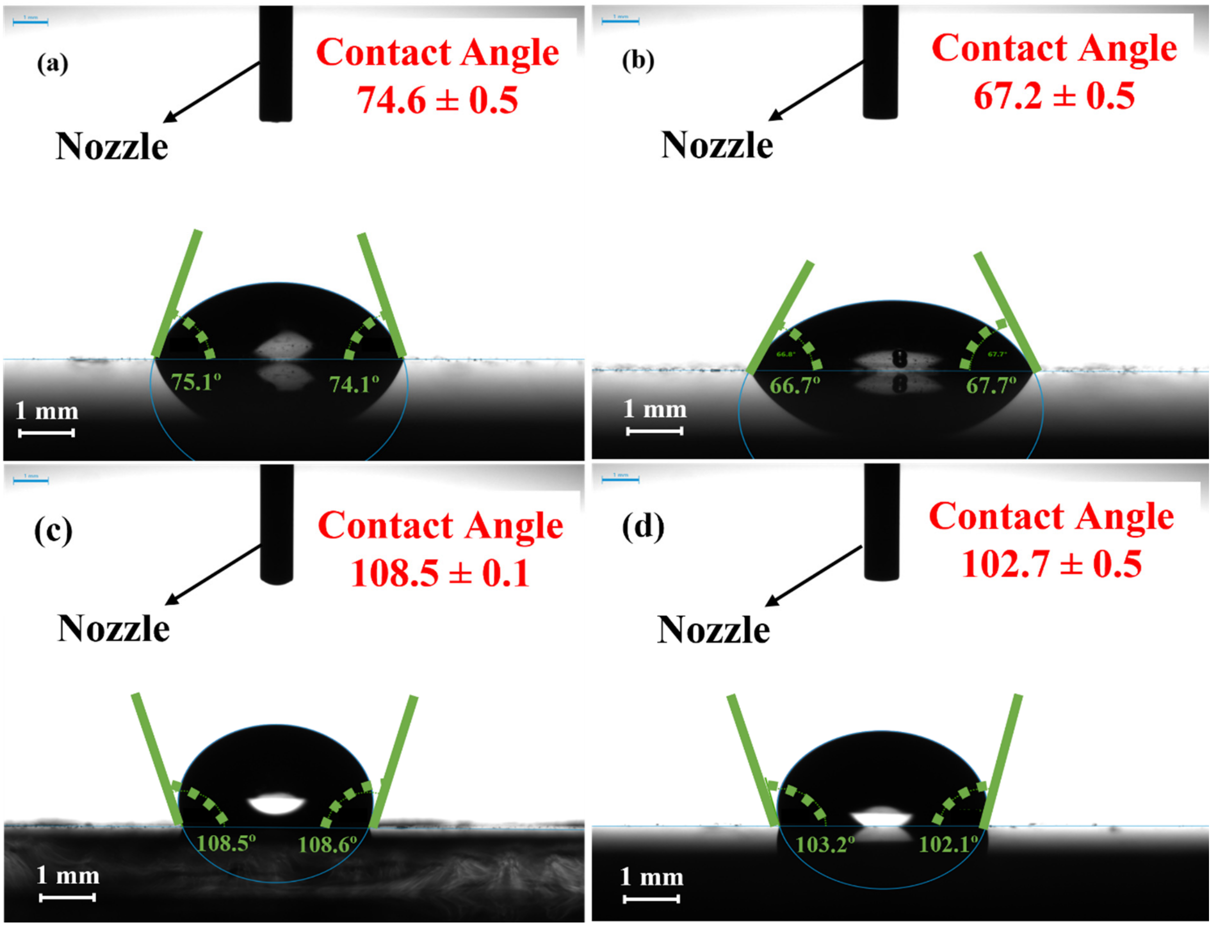

Figure S2. Contact angle images of substrates prepared with 1 ml sol (a, b) deposited with 20 and 30 cm spray distance at room temperature and (c, d) deposited with 20 and 30 cm spray distance at pre-heated substrates.

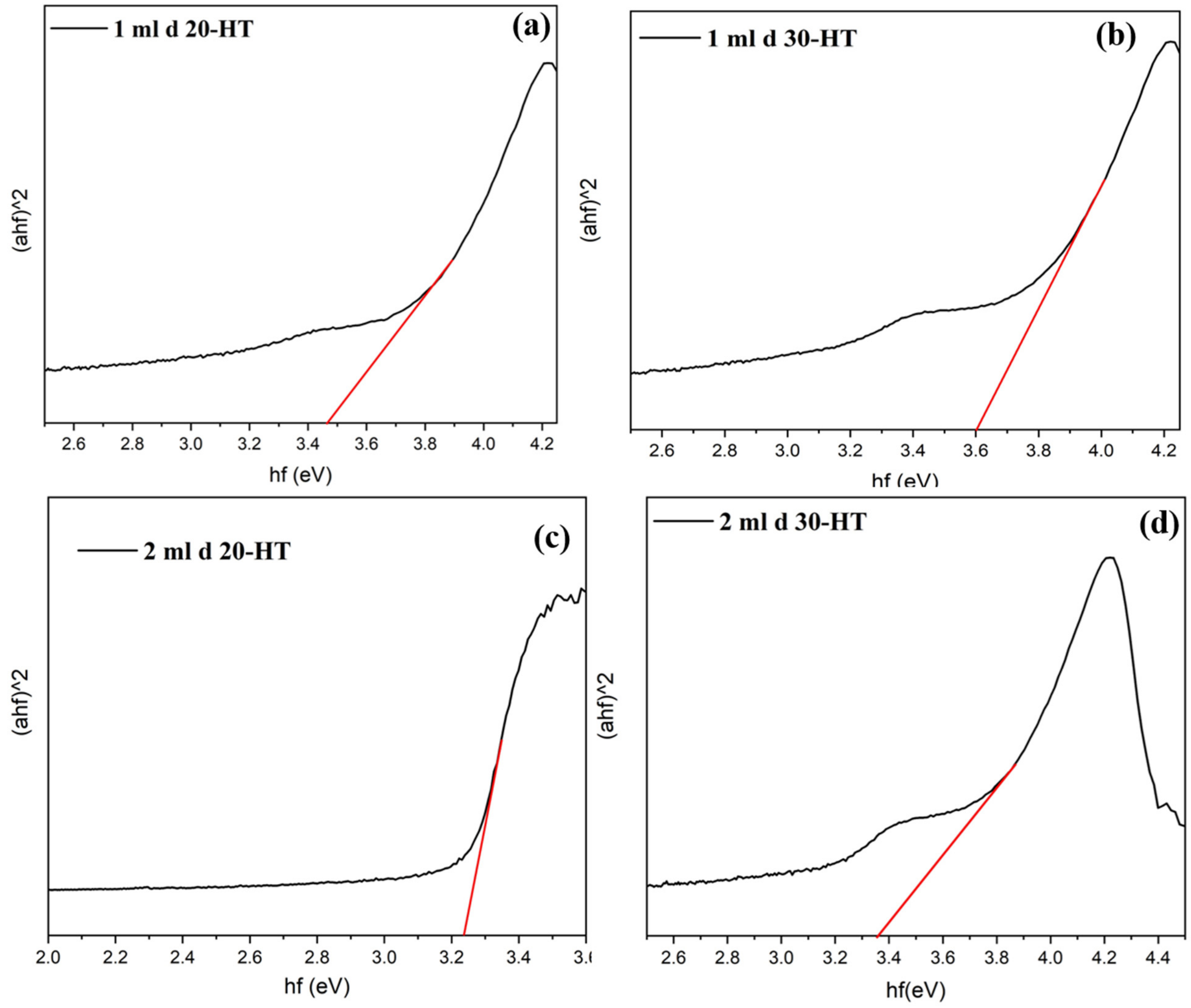

Figure S3. Bandgap of heated ZnO samples.
